# Acceptance of Illness and Health-Related Quality of Life in Patients After Myocardial Infarction—Narrative Review

**DOI:** 10.3390/jcm14030729

**Published:** 2025-01-23

**Authors:** Justyna Tokarewicz, Barbara Jankowiak, Krystyna Klimaszewska, Michał Święczkowski, Krzysztof Matlak, Sławomir Dobrzycki

**Affiliations:** 1 Department of Invasive Cardiology, Internal Medicine with CICU and Laboratory of Hemodynamics, Medical University of Bialystok, Jana Kilińskiego 1, 15-089 Białystok, Poland; 2 Department of Integrated Medical Care, Medical University of Białystok, Bialystok, Jana Kilińskiego 1, 15-089 Białystok, Poland; 3 Department of Cardiac Surgery, Medical University of Białystok, Bialystok, Jana Kilińskiego 1, 15-089 Białystok, Poland

**Keywords:** myocardial infarction, health-related quality of life, acceptance of illness, rehabilitation

## Abstract

**Introduction:** Cardiovascular diseases, particularly myocardial infarction (MI), significantly impact patients’ lives, causing stress and prompting varied responses to illness. **Aim and methods:** We conducted a narrative review concerning the acceptance of illness and quality of life in post-MI patients. Based on an extensive search of the available literature, this review consolidates current evidence on the proposed topic. **Conclusions and implications:** While some patients struggle with acceptance and face emotional distress, others who accept their condition are more likely to engage in treatment and lifestyle changes, leading to an improved health-related quality of life (HRQoL). Following an MI, patients often experience depression, anxiety, and stress, complicating their acceptance of the illness. Risk factors, such as hypertension, diabetes, and smoking, play a significant role in influencing HRQoL in post-MI patients. An accurate assessment of HRQoL is crucial for tailoring effective treatments and support strategies to enhance patient outcomes and identify those most at risk of developing post-MI depression or anxiety. Effective physician–patient and nurse–patient communication and support from family might be helpful in recovery. Cardiac rehabilitation improves patients’ outcomes and HRQoL. This review underscores the importance of integrating psychological support with optimal medical care to improve patient prognosis and enhance the HRQoL of individuals recovering from MI. The healthcare system could implement routine psychological assessments for MI patients at admission and discharge to establish a baseline for follow-up. Future research should explore effective psychological interventions, the interplay of CVD risk factors and psychosocial aspects, the emerging role of artificial intelligence in personalized care, and the cost-effectiveness of integrated treatment models.

## 1. Introduction

Cardiovascular diseases (CVDs) are the leading cause of mortality, morbidity, and disability, responsible for nearly 19 million deaths worldwide [1]. According to the Global Burden of Disease study, ischemic heart disease, which includes myocardial infarction (MI), is the leading cause of morbidity and mortality, responsible for over 315 million cases and 9 million deaths worldwide [2]. Myocardial infarction is the most severe, life-threatening presentation of coronary artery disease (CAD), and its prevalence among patients over the age of 60 years old is nearly 10% [3]. Almost 9% of patients under 45 years old experienced a new cardiovascular event during a median follow-up of less than 3 years [4]. Projections reveal a possible 30.1% increase in MI prevalence in the United States of America by 2060 [5]. Moreover, CAD has significant financial implications, with estimates suggesting that it cost the European Union over EUR 77 billion and resulted in the loss of approximately half a million working years in 2021 [6].

Experiencing an MI, particularly in young and professionally active individuals, might induce significant stress and trigger a sense of threat. Nevertheless, each person reacts to their illness differently, which is why the following two main groups of patients can be distinguished: those who accept their illness and those who do not. The acceptance of illness is the psychological adjustment to living with a medical condition, involving acknowledgement, adaptation, and resilience to maintain emotional stability and quality of life (QoL) [7]. Patients who do not accept their disease and experience unpleasant emotional distress caused by the illness are less likely to come to terms with it and make efforts to change their situation. Such individuals are more prone to feelings of sadness, anxiety, depression, and reluctance to maintain optimal health. On the other hand, those who accept their illness and the situation that they find themselves in take on the challenge of fighting the disease through consistent treatment, monitoring their health, acquiring knowledge about their condition, and making lifestyle changes, such as quitting harmful habits, modifying their diet, or incorporating physical activity into their life. Therefore, for patients who have experienced an MI and its complications, acceptance of their new situation is a very difficult and crucial process [8,9].

A critical aspect is the assessment of the health-related QoL (HRQoL) of patients after an MI. HRQoL refers to the subjective assessment of the impact of patients’ health status on their well-being. It encompasses aspects of physical, mental, and social–emotional functioning. HRQoL might include factors such as the ability to perform daily activities, pain levels, symptoms of depression and anxiety, and overall life satisfaction [10]. This is key to understanding the full impact of the disease on the daily functioning and overall well-being of patients. Improving the HRQoL for those affected by this disease requires a holistic approach that includes medical interventions, psychological support, social assistance, and lifestyle adjustments [11].

The objective of this narrative review is to examine the relationship between the acceptance of illness and the HRQoL in patients recovering from MI. By synthesizing current evidence, the review aims to identify the key factors influencing HRQoL, including psychological distress, risk factors, and the importance of effective communication and support systems. It also highlights strategies, such as cardiac rehabilitation and psychological interventions, to enhance recovery and improve patient well-being.

## 2. Literature Search Methods

**Figure 1:**
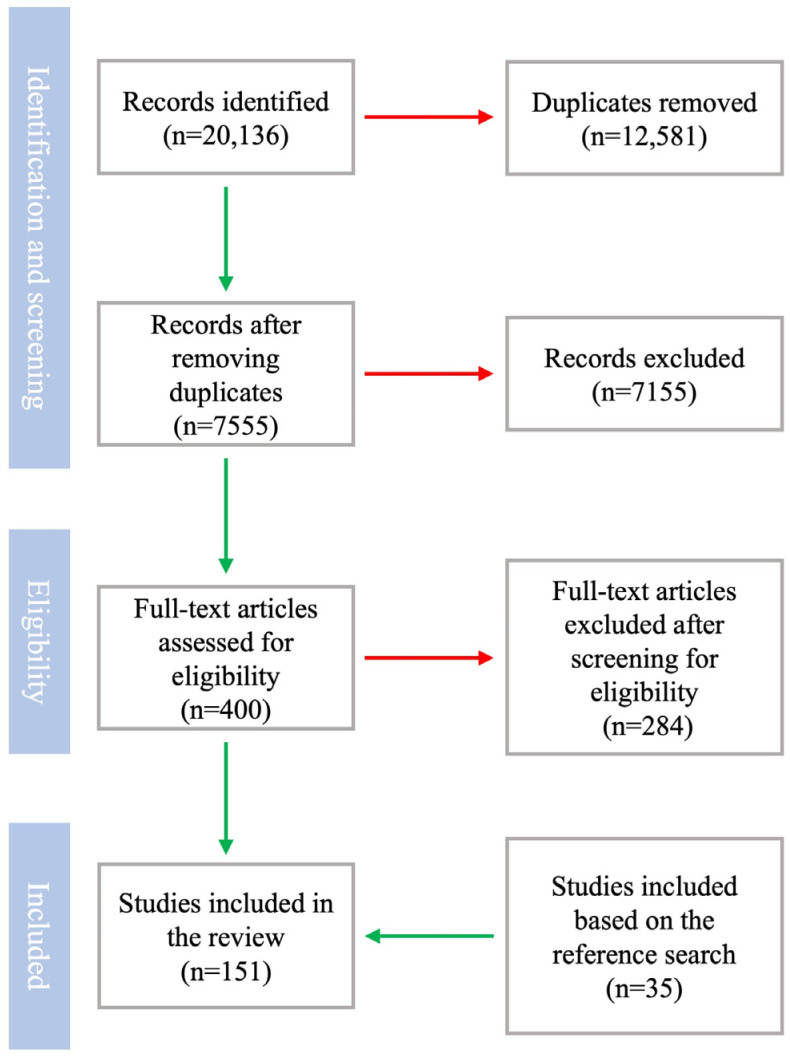
Literature search strategy.

PubMed, Scopus, Web of Science, Google Scholar, Embase, Cochrane Library, MEDLINE, and ResearchGate were used to search for articles that discussed the acceptance of illness and QoL in post-MI patients. The keywords and titles searched included “myocardial infarction”, “acute coronary syndrome”, “acceptance of illness”, “quality of life”, “health-related quality of life”, “hypertension”, “diabetes”, “air pollution”, “obesity”, “smoking”, “dyslipidemia”, “psychiatric disorders”, “quality of life questionnaires”, “cardiac rehabilitation”, “physician–patient communication”, “nurse–patient communication”, “wearables”, and “artificial intelligence”. Articles published up to December 2024 were considered. For each article considered as relevant, the reference list of eligible research was also browsed. The last literature search was conducted on 6 January 2024, resulting in 151 articles being included in the final version of the review. The eligibility for including a specific study in the review was assessed by two independent authors (JT and MŚ). A flowchart for the process of the identification, screening, and inclusion of articles is summarized in Figure 1.

## 3. Myocardial Infarction

According to the current fourth universal definition of MI, the term acute MI should be applied in cases of acute myocardial injury with clinical features of acute myocardial ischemia and biochemically confirmed myocardial cell necrosis. The standard marker of myocardial cell necrosis is currently the concentration of high-sensitivity cardiac troponin (cTn) in the blood, with at least one value exceeding the 99th percentile upper reference limit. Additionally, at least one of the following criteria must be met: symptoms of myocardial ischemia, new ischemic changes/pathological Q waves in the electrocardiogram, imaging evidence of a new loss of viable myocardium or new regional wall motion abnormalities, and the detection of coronary thrombus during coronary angiography or autopsy [12].

The initial clinical symptoms of an MI typically include chest pain or discomfort, often radiating to the upper limb (usually the left), jaw, or epigastrium, occurring during exertion or at rest. Infarction pain is typically burning, squeezing, or pressing in nature, and is usually intense, accompanied by fear for life. Additional symptoms include profuse sweating, shortness of breath, and fatigue. These symptoms occur regardless of body position. This is the typical presentation of MI pain; however, about 20% of MIs are painless, known as silent MI. Infarction pain is an alarming signal for patients, prompting them to seek medical help and offering the best chance for optimal treatment. An atypical course of a heart attack is often downplayed by patients, significantly delaying their response and prolonging the time to medical intervention. The first sign of an MI may be sudden cardiac arrest, which often occurs in young, previously healthy individuals. An atypical clinical presentation of MI, in the form of abdominal, pulmonary, or musculoskeletal symptoms, can delay diagnosis [13].

A quick and accurate diagnosis of MI through patient history, electrocardiogram, and blood cTn measurement is a key factor in making rapid treatment decisions and has a significant impact on a patient’s prognosis. Currently, modern treatment methods can reduce the extent of myocardial damage and prevent the development of heart failure [14,15].

## 4. Acceptance of Illness

In the face of an illness such as MI and its potentially severe complications that could lead to death, patients experience anxiety, which can lead to a lack of acceptance of the illness. This situation might trigger various emotional responses, ranging from denial and rejection to acceptance and assuming the role of a patient. Such a change in life can induce significant psychological discomfort and worsen adaptive abilities. The disease might generate immense stress and force individuals to make changes on multiple adaptive levels, which allow some degree of adjustment to the new and unfavorable situation. Consequently, a certain group of patients develop acceptance of their illness, which reduces the intensity of negative reactions and emotions and enables comprehensive treatment and care to be provided to these patients [7,16].

A patient’s attitude toward their illness depends on various factors, including personality, individual tendencies toward health behaviors, self-acceptance of the illness, stress coping strategies, and support from family, friends, and society [17]. In a new situation, such as the onset of disease, patients experience the emotion of loss. This involves a reduction in the ability to function effectively and a loss of familial, social, professional, and economic roles and interpersonal contacts due to the illness, its symptoms, complications, sometimes prolonged treatment, and therapy, as well as the fear of acquaintances witnessing pain and suffering [18]. The acceptance of illness is closely related to adaptation to the changes occurring in the body and daily life. It is a process occurring on both the emotional and cognitive behavioral levels. It involves adopting a positive attitude toward the existing situation, which motivates the patient to strive for recovery and prevents a decreased HRQoL during the illness [19].

Adaptation to illness is one of the most stressful events in a patient’s life and involves three stages related to the initial assessment of this stressful situation and the selection of stress-coping strategies. The first stage is acquiring the skills to manage the disease, which involves analyzing the initial experience of surprise and cognitive dissonance and waiting for a confirmation of the diagnosis. It is characterized by intense fear, anxiety, irritation, a sense of injustice and loss, and even a desire to escape. The second stage is seeking balance after the diagnosis is established. This is a period of reflection and searching for a way out of this difficult life situation. At this stage, many conflicting emotions and feelings may arise, ranging from a sense of injustice, loss, life failure, and doubt in the possibility of recovery to a positive outlook, where the illness is viewed as a challenge rather than an obstacle. The goal is to return to pre-illness life, with faith in the treatment offered and overcoming the disease. A strong desire to regain health might result in the activation of stress-coping strategies. The third stage is the release from habits and conditions associated with the disease. This period may involve resignation, surrender to the illness, and a withdrawal from life, with the predominance of an emotional stance towards the disease, or acceptance of the illness and active participation in the treatment process, with the predominance of a rational stance. The acceptance of illness is a complex process encompassing the emotional, cognitive, and adaptive spheres of patients living with a chronic illness, including MI [20].

To understand patients’ perceptions and management of illness, Leventhal’s model, known as the Self-Regulation Model or the Common-Sense Model, was developed. Key elements of this model include the representation of illness as identity, causes, duration, consequences, and control. Another element is the emotional processes that arise with the onset of illness, such as fear, anger, and depression, and their impact on coping with the illness and making treatment decisions. The next stage of the model involves coping strategies, which are methods that can be used to manage the disease and focus on both the problem and emotions. The final stage is evaluating the effectiveness of coping strategies and adjusting to the situation based on results and expectations. In Leventhal’s model, the acceptance of illness is a crucial element of the process, significantly influencing coping strategies and self-regulation [21].

## 5. Health-Related Quality of Life

In recent years, there has been significant interest in studying HRQoL as influenced by patients’ health conditions. Assessing the HRQoL in patients after an MI is essential for better understanding their needs and developing more effective treatment and support strategies. HRQoL for post-MI patients covers the several following aspects [22,23,24,25,26,27,28]:

- Physical health: This includes overall health status, access to medical care, and the quality of medical services received.
- Mental health: Defined by stress levels and emotional support.
- Material conditions: Including income, financial stability, and access to material resources.
- Social environment: Social relationships, support from family and friends, and involvement in social life.
- Housing conditions: Including the safety of the living environment.
- Education: Access to education and opportunities for intellectual development.
- Working conditions: Job security, job satisfaction, and work–life balance.
- Environmental factors: e.g., quality of air and water, green space availability.
- Culture and recreation: Access to cultural and recreational facilities and opportunities for leisure activities.

### 5.1. Risk Factors and Their Impact on Health-Related Quality of Life Post-Myocardial Infarction

Risk factors associated with CVD can significantly amplify the negative impact on the HRQoL of patients after experiencing an MI. This may be linked to, among other things, a lack of adequate education, changes in previous habits, and the need for frequent interactions with the healthcare system, as well as a high number of medications and their side effects. As the TIGRIS study revealed, a poorer HRQoL among post-acute coronary syndrome patients is associated with a higher risk of hospitalization and CVD events [29]. The main risk factors for CVD include hypertension, dyslipidemia, smoking, obesity, diabetes, and environmental burden. Assessing risk-factor-related HRQoL in hospital and post-discharge could be instrumental in determining which patients will benefit from tailored interventions.

#### 5.1.1. Hypertension

According to the 2024 ESC Guidelines for the management of elevated blood pressure and hypertension, hypertension is defined as a blood pressure (BP) level of 140/90 mmHg or higher [30]. Several studies have linked hypertension to a poorer HRQoL of patients [31,32,33,34,35]. Moreover, Schmieder et al. suggested that treatment-resistant hypertension might be associated with greater stress and anxiety than non-resistant hypertension, with 57% of respondents in this group reporting anxiety about managing their BP [36]. Trevisol et al. reported that hypertensive participants not using antihypertensive drugs had higher HRQoL scores than those on medication, regardless of their BP control status, suggesting that negative emotions related to medication use may play a role [32]. On the other hand, a study by Lambert et al. revealed that the HRQoL was lower in patients with significantly uncontrolled, treatment-resistant hypertension than those with controlled hypertension, with the former group exhibiting poorer scores in five out of eight SF-36 domains and the Mental Component Summary score [33]. The age of hypertensive patients affects their HRQoL, as elderly people were found to be more prone to adverse physical and psychological effects of the disease [37,38,39]. A lower economic status, self-management efficacy, and health literacy were also all linked to a poorer HRQoL in hypertensive patients [38]. According to Xiao et al., economic burden was the most common factor impacting patients’ HRQoL, with the suggestion that females might be more vulnerable when compared to males [40]. To improve the HRQoL of patients after an MI with hypertension, several potential interventions should be acknowledged. First, patients should be encouraged to undertake moderate physical activity [41]. Second, targeted intervention strategies, such as education and increasing health literacy and self-management efficacy, might improve drug compliance [37,38]. Third, increased access to free antihypertensive drugs, especially among older adults and those with a lower socioeconomic status, can improve the situation [38].

#### 5.1.2. Dyslipidemia

Dyslipidemia is defined by the 2019 ESC/EAS Guidelines for the management of dyslipidaemia as an abnormal level of blood lipids, specifically characterized by elevated low-density lipoprotein cholesterol (LDL-C), reduced high-density lipoprotein cholesterol, or increased triglycerides [42]. For all post-MI patients, the target LDL-C level is below 55 mg/dL or a 50% reduction, while for those with a recurrent CV event within two years, the target is as low as 40 mg/dL [43]. Statins are the drug of choice for hypercholesterolemia, with ezetimibe, bempedoic acid, and PCSK-9 inhibitors as alternatives in cases of intolerance or as an addition when therapeutic goals are not achieved. Wu et al. suggested that patients with a history of dyslipidemia had a poorer HRQoL (scoring significantly lower in four out of the five domains of the EQ-5D questionnaire), while Bahall et al. presented no such association [44,45]. HRQoL associated with dyslipidemia might be linked to the need for daily medication intake, with a lower HRQoL observed among patients who missed their daily doses or had more complex treatment regimens [46]. Moreover, Jarab et al. revealed that people who had concerns about dyslipidemia medications and received high-intensity statins or statins in combination with a fibrate had a lower HRQoL [47]. An observational study revealed suboptimal medication adherence among the Greek population with dyslipidemia, with 16% of patients not adhering to their prescribed lipid-lowering treatment, which had a detrimental impact on their HRQoL [48]. Researchers have emphasized that drug compliance significantly impacts hypercholesterolemic patients’ HRQoL, making proper education and simplifying treatment regimens critically important [47]. Additionally, new-generation drugs such as PCSK-9 inhibitors offer hope for improving the HRQoL in patients with hypercholesterolemia by allowing for less frequent dosing and providing a greater effectiveness [49]. Clinicians should devote more attention to non-pharmacological methods of dyslipidemia treatment, particularly for elderly people, focusing on a healthy lifestyle, diet, and physical activity [50,51].

#### 5.1.3. Smoking

The World Health Organization (WHO) describes smoking as the inhalation of tobacco products, which introduces harmful chemicals into the body and is highly addictive due to nicotine. Recognized as a leading cause of preventable disease, smoking results in over 8 million deaths per year, including more than a million fatalities among non-smokers exposed to second-hand smoke [52]. The decline in smoking rates has recently slowed across numerous countries, and while plans have been suggested to achieve tobacco-free generations, these measures have yet to be enacted [53]. Most of the observational studies conducted so far agree that there is a negative relationship between smoking and HRQoL [29,54,55,56]. Moreover, Buchanan et al. revealed that smoking after MI was associated with more symptoms and a lower HRQoL, while quitting smoking resulted in similar angina levels and mental health as never smokers [57]. Several studies have presented a positive impact of smoking cessation on overall HRQoL [54,55,56,57]. According to a meta-analysis by Hartmann-Boyce et al., behavioral support increased cessation rates by 44%, and the effect was slightly more visible without additional pharmacotherapy [58]. Post-MI smokers should be monitored closely and encouraged to cease smoking. Moreover, an individual approach appears to be insufficient, as systemic changes, such as increasing tobacco taxes, restricting the use of e-cigarettes, public health campaigns, age restrictions, and generational bans, should be considered in all countries, especially those struggling with high smoking rates.

#### 5.1.4. Obesity and Diabetes

Obesity and diabetes, key components of metabolic syndrome, are not only major risk factors for CVD, but also significantly impact HRQoL, especially in patients following MI. Obesity, defined as a body mass index (BMI) of ≥ 30 kg/m^2^, was declared a ‘global epidemic’, underscoring its rapid rise and the widespread health risks it poses across all age, sex, and socioeconomic groups [59]. On the other hand, approximately 529 million people were living with diabetes worldwide in 2021, and by 2050, more than 1.3 billion people are projected to have diabetes [60]. In a systematic review of 12 meta-analyses and reviews, obesity was associated with a significantly lower generic and obesity-specific HRQoL [61]. Donini et al. identified BMI, disability, and psychological symptoms as the main determinants of HRQoL in obese patients [62], whereas Abiri et al. reported a higher risk in obese patients with an adverse metabolic profile, such as comorbid diabetes [63]. Moreover, survivors of MI with coexisting diabetes experienced a reduced HRQoL at discharge from the hospital, and the presence of long-term health conditions further diminished their likelihood of improvement [64]. Similar results were published by Kang et al., in which diabetes was negatively correlated with HRQoL [65]. Furthermore, comorbid chronic diseases and a low socioeconomic status had adverse effects on the HRQoL in diabetic patients [66]. A reduced HRQoL might be associated with a high frequency of oral medication or insulin use, the costs involved, and the multiple comorbidities that often coexist with diabetes. Firstly, clinicians should prioritize encouraging patients to use non-pharmacological methods for weight reduction and glycemic control, such as diet and moderate physical activity [67]. Secondly, modern medications like GLP-1 analogs (e.g., tirzepatide) have shown effectiveness in treating both obesity and type 2 diabetes [68]. Thirdly, there are surgical options for managing obesity, as recent research has consistently demonstrated a positive relationship between weight loss and an improved HRQoL following bariatric surgery [61]. Finally, a new generation of insulin, ‘icodec’, administrated only once weekly, could simplify treatment regimens [69].

#### 5.1.5. Environmental Factors

The main environmental risk factor is air pollution, which contributes to the development of various CVDs, including MI and ischemic stroke [70,71,72,73,74]. Initial reports on the adverse effects of noise and light pollution are also emerging, although the level of evidence remains weak [75]. According to a recent ECRHS study, Europeans residing in areas with high concentrations of air pollution and low levels of greenspace were more likely to have lower Mental Components Summary (MCS) scores on SF-36, with each unit increase in PM_2.5_ associated with a 1.79 decrease in MCS [76]. Moreover, short-term exposure to carbon monoxide and sulfur dioxide was associated with lower EuroQol-visual analog scale scores among the Korean population, with reductions of −1.571 per interquartile range increment in carbon monoxide and −1.722 per interquartile range increase in sulfur dioxide [77]. Household air pollution also plays a significant role, especially in low- and middle-income countries, as exposure to this kind of pollution had a negative correlation in a Chinese study [78]. As proposed by the 2021 ESC Guidelines on cardiovascular disease prevention in clinical practice, patients at a high or very high CVD risk should be encouraged to avoid long-term exposure to air pollution [79]. Moreover, in areas with high levels of air pollution, CVD risk screening programs might be considered [79]. The same document highlights the need for systemic changes, such as lowering particulate emission and gaseous pollutants, reducing the use of fossil fuels, and limiting carbon dioxide emissions [79].

In summary, comorbidities significantly impact the HRQoL of patients, including those post-MI. Evidence reveals that adherence to secondary prevention post-MI remains very low, with the most vulnerable groups being females, diabetic patients, obese people, and patients with chronic kidney disease or depression [80,81].

## 6. Psychological Support

Anxiety, fear, and even depression can be integral components during the onset of a disease. These emotions might significantly impact a patient’s HRQoL. Table 1 presents the prevalence of psychiatric disorders among MI survivors.

Studies have shown that the prevalence of depression shortly after an MI ranges from 13% to 66%, with symptoms often persisting during follow-up periods [82,83,84,85,86,87]. Several studies have linked depression with worse outcomes in cardiovascular patients [84,88]. The early days following the event appear to be crucial. Murphy et al. recognized factors associated with an increased risk of depression and anxiety, such as a history of depression, financial difficulties, a poor self-assessed health, a lower socioeconomic status, a younger age, and smoking [9]. Other studies have identified obesity/diabetes [89], social isolation [90], and a lack of marital status [91].

In summary, patients who have experienced an MI are exposed to significant emotional stress, increasing their risk of developing depression and anxiety. Identifying at-risk individuals early during hospitalization provides an opportunity to offer support and potentially prevent future mental health challenges. Furthermore, early intervention might enhance the QoL for these patients.

## 7. Quality of Life Questionnaires

QoL questionnaires are tools used to assess the subjective perception of well-being by patients, encompassing various aspects of life such as physical health, mental health, social relationships, work, and overall satisfaction with daily life. Numerous QoL questionnaires exist, utilized both in scientific research and clinical practice. These can be categorized into general questionnaires (Table 2), applicable across various diseases and population groups, and disease-specific questionnaires (Table 3) designed for specific medical conditions. Using these tools in patient care might help to evaluate the impacts of diseases and treatments on patients’ daily functioning.

## 8. Communication

### 8.1. Physician–Patient Communication

Effective communication between physicians and patients is a cornerstone of post-MI care. Over the last decades, a shift from physician-centered to patient-centered care has been observed in the physician–patient relationship, increasing the odds of effective and competent communication. It promotes mutual understanding between physicians and patients, thus allowing patients to take a more active role in the therapeutic process. The key components of patient-centered communication include sharing information, supporting patients in self-management, addressing uncertainty and emotional concerns, supporting decision making, and fostering a strong physician–patient relationship [102]. According to Jacquelin et al., the central aspects of effective physician–patient communication are clear and explicit language, patient participation and activation, negotiating epistemic knowledge, affiliative language and building emotional bonds, role and identity, and managing the interactional and relational aspects of communication [103]. Lerch et al. suggested that the factors influencing communication included patient-related aspects such as psychological factors, health education, literacy levels, and the social environment, while physician-centered elements that contributed to a trusting patient–physician relationship encompassed competence, effective communication, genuine interest in the patient, caring, health education, and professionalism [104]. Cuevas et al. reported lower physician mistrust in patients exposed to highly patient-centered communication than among patients exposed to low patient-centered communication [105].

Post-MI care typically involves complex medical regimens, including medications, lifestyle modifications, and follow-up appointments. A meta-analysis of 127 studies revealed a correlation between high-quality communication and better adherence to therapy, as there was a 19% higher risk of non-adherence among patients whose physicians communicated poorly [106]. Moreover, there was a 12% higher risk of non-adherence among patients with physicians who had not been trained in communication skills [106]. Izumi et al. reported that patient satisfaction depends on communication with the physician [107]. Physician empathy significantly affected outcomes (41% lower rates of acute metabolic complications) for patients with diabetes mellitus, an important risk factor of MI [108]. Scientists debate the impact of certain variables on patient trust; some argue that the characteristics of the patient and physician (such as shared race [109]) significantly influence it, while others claim that these characteristics do not matter [108]. However, physician burnout may play a significant role, as physicians are particularly vulnerable to it, and research suggests that it has a substantial impact on patient satisfaction [110].

In summary, implementing individual and organizational interventions can effectively reduce burnout levels among physicians, therefore improving physician–patient communication [111]. According to Amutio-Kareaga et al., practicing mindfulness may counteract this process by fostering self-awareness and emotional regulation skills, which help physicians to become more receptive to patients’ perspectives and demonstrate greater empathetic concern [112]. Furthermore, training and targeted interventions that emphasize the importance of effective communication may be crucial in improving adherence and, consequently, the HRQoL of patients, including those post-MI. Lastly, there is still a need for research on the factors influencing patient adherence and physician–patient communication, as well as the impact of both on patients’ HRQoL post-MI.

### 8.2. Nurse–Patient Communication

Nurse–patient communication during illness might play a significant role in helping patients to accept their new health condition. This process is comprehensive and multifaceted, encompassing medical and psychological dimensions [113]. The nurse–patient and nurse–family relationships are critical in ensuring safety, comfort, and emotional support for both the patient and their family. Similar to physician–patient communication, a patient-centered approach is preferred [114].

Education plays a pivotal role in the nurse–patient and nurse–family relationships, forming an essential element of medical care. In a nurse-led individualized self-care model, nurses collaborate with patients to set realistic goals based on the specific characteristics of the patient’s condition, implementing tailored nursing programs designed to encourage lifestyle changes that enhance their overall health. A nurse-led lifestyle modification follow-up program in India significantly improved both systolic and diastolic blood pressure, body mass index, and HRQoL in all domains (physical, emotional, and social) after 12 weeks of discharge in patients post-MI [115]. Zhao et al. demonstrated that the nurse-led individualized self-care model significantly improved HRQoL, self-care ability, health behaviors, self-efficacy, and glycemic control in post-MI patients with type 2 diabetes compared to standard care [116]. Moreover, in the KORINNA Follow-Up Study, nurse-based case management intervention in elderly patients increased the HRQoL in the intervention group among MI survivors after 3 years [117]. A multicenter study showed that a nurse-led approach in primary care during the initial months post-MI might enhance adherence to diet, medication, and physical activity [118]. Making patients aware of the beneficial actions they can take reduces hospital readmissions and outpatient visits, contributing to the efficiency of the healthcare system.

There is also a psychological aspect to this relationship, as nurses often assume the role of psychotherapists, supporting patients and their families in coping with the emotional impact of life-threatening conditions such as MI. For example, patients with scheduled counselling by qualified nurses the day before and 24 h after a percutaneous coronary intervention (PCI) procedure were less anxious compared to groups with standard pre-procedural information [119]. Jiang et al. presented similar conclusions, as a nurse-led individualized self-management program for patients with MI undergoing PCI resulted in better risk factor control and a higher HRQoL at 12-month follow-up [120]. On the other hand, there are several conflicting reports. In a randomized, controlled trial of 1376 post-MI patients, a home-based psychosocial nursing intervention did not affect prognosis or anxiety symptoms, although the study group was unbalanced in terms of the participants’ sex, and women, who were in the minority, exhibited significantly worse survival rates [121]. Furthermore, a nurse-led psychological intervention in intensive care unit patients did not significantly influence patient-reported post-traumatic stress disorder symptom severity at 6 months follow-up [122]. However, the studied population consisted of critically ill patients, so these results cannot be easily extrapolated to other patient groups with milder conditions [122].

In summary, guidance by nurses might improve health outcomes, HRQoL, and adherence to treatment in post-MI patients. Moreover, the nurse–patient relationship often extends to psychological support; conflicting evidence highlights the need for tailored interventions and balanced study designs to fully assess their impact.

## 9. Role of Family

Family is one of the most crucial environments for an individual, often providing essential support for their life and development. Observational studies suggest that impairments in family functioning, such as reduced cohesion and interpersonal conflicts within family relationships, are significant risk factors for the development of an MI [123]. The family’s attitude and behavior play significant roles in the process of a patient’s acceptance of their illness. A supportive stance that fosters a sense of security, understanding, and acceptance significantly influences how the patient copes with the diagnosis, treatment, and daily challenges arising from the illness. From the initial diagnosis, managing the new situation, medical appointments, and medication use to the therapeutic process, these typically occur within the family and through its mediation. As was presented by Asgari et al., including a family-centered education model improved the laboratory results of post-MI patients [124]. In a clinical trial, organizing a workshop through multimedia software for post-MI patients and their family members for only one month was associated with a higher HRQoL [125]. Moreover, involving the family in cardiac rehabilitation was proven to encourage physical-activity-related interactions [126].

In the event of an illness affecting one family member, the normal life of the entire family can become disorganized [127]. The impact of the illness on the family depends on the nature of the disease. A sudden onset of illness in a close family member, particularly in the case of an MI, is a source of stress, whereas a gradual progression of the disease is less burdensome, as it allows more time for the family to adapt to the situation. This process, referred to as adaptation, provides the family with an opportunity to become accustomed to the illness. The course of the disease, which creates a constant sense of threat to the family, significantly influences their behavior [128].

The impact of illness on a family depends on its developmental stage, the presence or absence of its members, the quality of the relationships among them, the availability of support, the level of religiosity within the family, and their beliefs about the causes and meaning of the illness. The family’s approach to illness and their ability to manage it also depends on their conceptual framework of family functioning. In the linear model of relationships and interdependencies within a family, the theory of family burden is applied [127]. In cases of illness, families are confronted with objective burdens, such as financial costs, social relationship disruptions, and limitations in personal activities, as well as subjective burdens, including the emotional strain and attitudes of family members caring for the ill individual. This theory focuses on the negative consequences of caregiving, often causing the patient to feel like a “burden” to their family and friends. In contrast, the systemic family model emphasizes the interactions and dynamics among family members [127]. Adaptation, central to this model, refers to the family’s adjustment to the challenging situation posed by the illness of one of its members. The goal is to meet the needs of all members and enable the family to function effectively within society. This model focuses on solving problems and coping with challenges through specific strategies, such as sharing concerns with other family members, rational thinking, temporary distraction through engagement in other activities, and acceptance of the illness [127].

## 10. Cardiac Rehabilitation

The development of new medications, medical devices, and coronary interventions has significantly improved the clinical prognosis for patients after an MI over recent decades. The primary goal of clinicians and researchers is to reduce mortality and the incidence of serious cardiovascular events. However, particularly in low- and middle-income countries, there remains a lesser emphasis on post-hospital care for patients. As Hammer et al. revealed, only 13.4% of patients after MI adhered to the recommended cardiac rehabilitation programs [129]. Recent studies suggest that physical and psychological rehabilitation following a cardiovascular incident can significantly enhance patients’ HRQoL (Table 4). Furthermore, researchers highlight gender disparities, indicating that women are more likely to experience a poorer HRQoL after an MI. Consequently, it is crucial to pay particular attention to specific social groups and tailor rehabilitation methods to their needs [27].

## 11. Future Directions

The healthcare system should prioritize integrating psychological support into MI treatment protocols. This support should be a standardized part of both inpatient care and outpatient follow-up, emphasizing the importance of mental health in physical recovery. A randomized clinical trial demonstrated that early, comprehensive psychological intervention significantly reduced anxiety and depression rates while enhancing HRQoL compared to the control group [137]. Hospitals could introduce routine psychological assessments for MI patients upon admission and before discharge, creating a baseline for continued follow-up. Williams et al. suggested routine screening for depression in post-MI patients [138]. Policies that ensure access to psychological support in follow-up care through affordable counselling sessions and community-based support groups can make these services accessible and sustainable. The family members of patients should have access to appropriate training, as, according to research, peer support can be used as a complementary method to improve post-MI patients’ HRQoL [139].

The 21st century is marked by rapid advancements in technology, including artificial intelligence, which can be utilized to improve HRQoL after an MI. Johnston et al. presented that using smartphone applications aimed at improving treatment adherence and cardiovascular lifestyle in MI patients significantly increased adherence (6.2 lower non-adherence score) and satisfaction (9.2 higher system usability score) compared to the control group [140]. Similar, promising results were presented by Varnfield et al., where a smartphone-based home care cardiac rehabilitation program improved post-MI cardiac rehabilitation uptake, adherence, and completion [141]. Moreover, implementing mHealth-CR had a substantial impact on enhancing both inner strength and resilience in elderly patients after an MI [142]. Wearable devices offer a valuable, modern solution that can serve as an alternative or complement to stationary rehabilitation for patients with coronary artery disease [143]. Wearable activity trackers significantly increase physical activity in various age groups, equating to 1800 additional steps per day, 40 extra minutes per day more walking, and a loss of approximately 1 kg in body weight [144]. Emerging technologies such as artificial intelligence and wearable devices are promising not only for improving recovery post-MI, but also play a significant role in empowering patients to manage their health proactively, e.g., arrhythmia assessment. They can significantly aid post-MI recovery by offering real-time monitoring and promoting healthy behaviors. Wearable activity trackers can help patients to adhere to exercise regimens by tracking steps, heart rate, and progress, whereas smartphone applications might provide medication reminders and educational resources. Additionally, virtual rehabilitation programs provide accessible, personalized care, especially for patients unable to attend stationary rehabilitation. However, despite the potential of these emerging technologies, several barriers such as limited access to technology, lack of digital literacy, privacy concerns, and financial constraints should be considered, which might hinder their widespread implementation, particularly among elderly and socioeconomically disadvantaged populations. Addressing these challenges is essential for maximizing the impact of these technologies on improving the HRQoL of post-MI patients.

Future research should focus on identifying the specific psychological interventions that are the most effective in improving illness acceptance and HRQoL in post-MI patients. More prospective studies should investigate the interplay between CVD risk factors (e.g., hypertension and diabetes) and psychosocial factors in shaping patient outcomes. Examining the role of emerging technologies, such as the use of AI and digital health tools, in tailoring personalized cardiological and psychological care could provide valuable insights. Investigating the cost-effectiveness of integrated care models that combine optimal medical treatment, psychological counselling, and social support would also be valuable. Moreover, there is a need for research into new pharmacotherapies that not only improve the prognosis of MI patients, but also minimize the risk of complications and enhance adherence to therapy, which could collectively lead to a better HRQoL. An especially effective solution regarding drug compliance is the use of polypills. As the recent SECURE trial revealed, there was a significant 24% reduction in cardiovascular events and death among polypill users in comparison to usual care in MI patients [145]. In the UMPIRE trial, the use of polypills resulted in improvement in drug compliance and risk factor control [146]. On the other hand, in studies by Patel et al. and Selak et al., there was greater adherence to the recommended therapy in the polypill group compared to the control group, although the clinical difference was not significant between the two analyzed groups [147,148]. New drugs may also contribute to improving prognosis, adherence, and HRQoL. For example, SGLT-2 inhibitors, indicated for type 2 diabetes, chronic kidney disease, and heart failure, have also shown potential applications in reducing contrast-induced acute kidney injury rates in patients with acute coronary syndromes undergoing PCI [149]. Despite their proven benefits in these indications, existing studies have not demonstrated a prognostic benefit in acute coronary syndrome settings [149]. Moreover, GLP-1 analogs, such as tirzepatide, have proven effective in managing both type 2 diabetes and obesity, especially in high-risk CVD patients [68]. In the FINEARTS-HF trial, finerenone was shown to reduce the risk of primary outcomes and improve symptoms in patients with heart failure with mildly reduced or preserved ejection fraction [150]. In the future, gene therapy may play a key role in treating cardiovascular diseases, as therapies like zilebesiran, an RNA interference-based agent, can significantly lower blood pressure for up to 24 weeks with just one subcutaneous dose and minimal side effects [151]. Summary of most critical studies included in this review is presented in Table S1.

In summary, early psychological interventions for patients after an MI should become standard practice. Modern, interactive solutions may serve as an alternative or complement to traditional approaches in supporting these patients, although several barriers in implementation have to be considered. Additionally, further research is essential to better understand the topic and refine approaches to improving outcomes in post-MI patients, including the development of new drug treatments and gene-based approaches, which have shown promising results.

## 12. Conclusions

MI is a life-threatening condition and might significantly impact patients’ HRQoL. Comorbid risk factors such as hypertension, diabetes, and smoking can further decrease patients’ HRQoL. The acceptance of illness is crucial in the process of adaptation and it might aid in better management of the disease. Effective physician–patient and nurse–patient communication might improve patients’ drug adherence, outcomes, and, therefore, their HRQoL. The routine use of QoL questionaries can help to identify early the patients most at risk of developing psychiatric disorders such as depression and anxiety. Cardiac rehabilitation (including the use of modern solutions such as artificial intelligence and digital health technologies), health education, and appropriate psychological treatment are essential for improving HRQoL in patients post-MI. Policymakers should advocate for integrating mental health care into routine cardiovascular care, promoting widespread access to psychological services and interventions. Initiatives aimed at increasing public awareness of the mental health challenges associated with MI could encourage early help-seeking behaviors among patients. There is still a need for continued research into the psychological aspects of post-MI recovery, personalized interventions, its cost-effectiveness, and the integration of emerging technologies to optimize patient care. Addressing the gaps in research on adherence, physician–patient communication, and the psychological impact of comorbidities will be critical to improving the HRQoL and long-term outcomes for MI patients.

## Figures and Tables

**Table 1:** Selected studies on the prevalence of psychiatric disorders following MI.

Authors	Year of Publication	Group	Psychiatric Disorders Prevalence	Conclusion
Kala et al. [82]	2016	79 patients with STEMI treated by pPCI	24 h: 21.5% depression, 8.9% anxiety; 3–5 days: 8.9% depression, 0% anxiety; 3 months: 10.4% depression, 4.5% anxiety; 15.4% depression, 10.8% anxiety; 1 year: 13.8% depression, 6.2% anxiety	A noticeable reduction in mental stress was observed prior to hospital discharge; however, within a year following PCI, the prevalence of both symptoms gradually rose.
Hanssen et al. [83]	2009	288 MI patients	Baseline: 13.6% depression, 19.7% anxiety; 3 months: 13.4% depression, 16.1% anxiety; 6 months: 14.7% depression, 16.5% anxiety; 12 months: 10.2% depression, 14.1% anxiety; 18 months: 13.7% depression, 16.8% anxiety	In the early phase, many MI patients experience psychiatric disorders, however, long-term prevalence of anxiety and depression is similar to general population.
Wheeler et al. [84]	2012	337 MI patients	Baseline: 39.3% depression	Moderate to severe depression was associated with higher all-cause mortality at 5-years follow up.
Johansson et al. [85]	2010	204 MI patients	4 months after MI: 20% depression, 25% anxiety	It is important to acknowledge psychosocial symptoms and needs due to their significant impact on health and overall well-being.
Parashar et al. [86]	2009	2411 MI patients	Baseline: 22.3% depression (29% in females, 18.8% in males)	Higher prevalence of depression after MI in females. Depressive symptoms are predictors of worse outcomes in men and women following an MI, with a comparable level of impact.
Hosseini et al. [87]	2009	806 MI patients	Baseline (within 15 days after the event): 65.9% depression, 69% anxiety	Very high prevalence of depression and anxiety in the studied population, however, it did not affect cardiac outcomes.

Abbreviations: MI, Myocardial Infarction; pPCI, Primary Percutaneous Coronary Intervention; STEMI, ST-Elevation Myocardial Infarction.

**Table 2:** Selected general quality of life questionnaires for patients.

Questionnaire, Year of Development	Characteristics
Short-Form Health Survey 36 (SF-36), 1992 [92]	36 questions assessing 8 domains: physical functioning, role physical, bodily pain, general health, vitality, social functioning, role emotional, and mental health; widely used across various populations and clinical conditions.
World Health Organization Quality of Life—BREF (WHOQOL-BREF), 1996 [93]	26 questions assessing 4 domains: physical health, psychological health, social relationships, and environment.
EuroQol-5D (EQ-5D), early 1990s; EQ-5D-3L in 1990, EQ-5D-5L in 2009 [94]	5 questions covering mobility, self-care, usual activities, pain/discomfort, and anxiety/depression, along with a visual analog scale (VAS) for overall health assessment.
Nottingham Health Profile (NHP), 1979 [95]	Two-part questionnaire. The first part assesses six areas: energy, pain, emotions, sleep, social isolation, and physical limitations, while second part focuses on problems with daily functioning.
Sickness Impact Profile (SIP), 1975 [96]	Assesses the impact of illness on various life aspects, such as mobility, communication, daily activities, emotional, and social functioning.
Satisfaction with Life Scale (SWLS), 1985 [97]	Consists of 5 questions rated on a scale from 1 (strongly disagree) to 7 (strongly agree). The questions address aspects such as satisfaction with life, achievements, and future outlook.

**Table 3:** Selected disease-specific quality of life questionnaires for CVD patients.

Questionnaire and Year of Development	Characteristics
Seattle Angina Questionnaire (SAQ), 1995 [98]	Measures the QoL in patients with coronary artery disease. Consists of 19 questions addressing angina symptoms, functional status, and QoL.
Kansas City Cardiomyopathy Questionnaire (KCCQ), 2000 [99]	Assesses the QoL in patients with heart failure (HF). Comprises 23 questions evaluating symptoms, activity limitations, functional status, and overall health perception.
Cardiac Depression Scale (CDS), 1996 [100]	Assesses depression and QoL in patients with cardiac conditions. Includes questions about depressive symptoms that may impact the QoL.
The HeartQoL, 2014 [101]	14-item questionnaire to assess QoL in patients with ischemic heart disease, consisting of 10 physical and 4 emotional items.

**Table 4:** Physical and psychological rehabilitation post-myocardial infarction.

Authors	Year of Publication	Study Type	N of Patients	Conclusions
Campo et al. [130]	2020	RCT	235	Exercise intervention improves mobility, daily activities, HRQoL, and outcomes in older patients with ACS.
Peixoto et al. [131]	2015	RCT	88	Cardiac rehabilitation program improved HRQoL and functional capacity in patients at low cardiovascular risk after MI.
Hurdus et al. [132]	2020	Multicenter observational	3438	Cardiac rehabilitation was associated with improved HRQoL at up to 12 months.
Prabhakaran et al. [133]	2020	RCT	3959	Yoga-based cardiac rehabilitation improved self-rated health and return to pre-infarct activities after MI.
Pristipino et al. [134]	2019	RCT	94	Psychotherapy after MI improves clinical outcomes overall up to 5 years post the incident.
Blumenthal et al. [135]	2016	RCT	151	Adding stress management training to routine cardiac rehabilitation significantly reduced stress and improved medical outcomes.
Liu et al. [136]	2024	Single-center observational	94	Integrating the 5E rehabilitation nursing model including mindfulness training significantly decreases stress, improves HRQoL and satisfaction in MI patients after PCI.

Abbreviations: ACS, Acute Coronary Syndrome; HRQoL, Health-Related Quality of Life; MI, Myocardial Infarction; N, Number; PCI, Percutaneous Coronary Intervention; RCT, Randomized Controlled Trial.

## Data Availability

Not applicable.

## Supplementary Materials

The following supporting information can be downloaded at: https://www.mdpi.com/article/10.3390/jcm14030729/s1, Table S1. Summary of most critical studies included in this review.
