# Supplementary material for: Acceptance of Illness and Health-Related Quality of Life in Patients After Myocardial Infarction—Narrative Review
Source: J Clin Med. 2025 Jan 23;14(3):729. doi: 10.3390/jcm14030729 (PMC11818487; doi:10.3390/jcm14030729)
Supplement: Supplementary file 1 [file jcm-14-00729-s001.zip › jcm-3216271-supplementary.pdf]

Acceptance of illness and health-related quality of life in pa-tients after myocardial infarction – a narrative review

Justyna Tokarewicz, Barbara Jankowiak, Krystyna Klimaszewska, Michał Święczkowski, Krzysztof Matlak and Sławomir Dobrzycki

Supplementary materials:

Supplementary Table S1. Summary of most critical studies included in this review.

Authors	***Title***	***Year of publication***	***Type of the study***	***Conclusions***
Dabek et al. [8]	Quality of Life in Patients with Coronary Artery Disease-Multicenter POLASPIRE II Study	2024	Multicentre, observational study	Quality of life (QoL) is influenced by factors such as gender, body weight, and adherence to medical recommendations, emphasizing the necessity for improved patient education to promote healthy lifestyle choices and enhance both life quality and longevity.
Leventhal H et al. [21]	The Common-Sense Model of Self-Regulation (CSM): a dynamic framework for understanding illness self-management	2016	Review	The Common-Sense Model of Self-Regulation provides a dynamic framework for understanding how patients perceive, manage, and respond to health threats, and emphasizes the need for future research to fully explore the model's adaptive components to enhance medical practices and illness self-management.
Lichtman JH et al. [26]	Depression as a risk factor for poor prognosis among patients with acute coronary syndrome: systematic review and recommendations: a scientific statement from the American Heart Association	2014	Systematic review	Despite the variability among studies, the evidence strongly supports the recommendation to recognize depression as a risk factor for adverse medical outcomes in patients with acute coronary syndrome, highlighting its critical role in patient prognosis.
Pocock S et al. [29]	Health-related quality of life 1-3 years post-myocardial infarction: its impact on prognosis	2021	Multicentre, observational study	A lower health-related QoL (HRQoL) in patients following acute coronary syndrome is associated with an increased likelihood of hospital admissions, major cardiovascular events, and death, indicating the importance of addressing HRQoL in post-acute care management.
Wang W et al. [34]	Health-related quality of life and its associated factors in Chinese myocardial infarction patients	2014	Observational study	Evaluating the HRQL of myocardial infarction (MI) patients and identifying the factors involved can assist healthcare professionals in developing interventions that are both tailored and culturally appropriate for this patient population.

Wu H et al. [44]	Evaluation of health-related quality of life in adults with and without dyslipidaemia in rural areas of central China	2020	Observational study	Dyslipidemia was linked to lower HRQoL, with factors like age, sleep quality, non-communicable diseases, and depression serving as significant predictors, while many patients remained unaware of the risks associated with an unhealthy lifestyle.
Cesaro et al. [49]	Impact of PCSK9 inhibitors on the quality of life of patients at high cardiovascular risk	2020	Prospective, observational study	Proprotein convertase subtilisin/kexin type 9 inhibitors (PCSK9 inhibitors) improve the QoL and overall health status in patients at high or very high cardiovascular risk, with effects extending beyond just lowering low-density lipoprotein cholesterol (LDL-C) and positively influencing prognosis.
Wu AD et al. [56]	Smoking cessation for secondary prevention of cardiovascular disease	2022	Systematic review	Smoking cessation reduces the risk of cardiovascular disease (CVD) death, major adverse cardiovascular events (MACE), and improves QoL, with moderate certainty for CVD death and low certainty for MACE, suggesting significant health benefits from quitting smoking.
Kuzma et al. [71]	Effect of air pollution exposure on risk of acute coronary syndromes in Poland: a nationwide population-based study (EP-PARTICLES study)	2024	Observational study	Air pollution exposure significantly increases the risk of MI, particularly for younger individuals, women, rural residents, and those with lower incomes.
Boudier et al. [76]	Long-term air pollution exposure, greenspace and health-related quality of life in the ECRHS study	2022	Observational study	European adults living in areas with higher air pollution and less green space are more likely to have lower mental QoL.
Kala et al. [82]	Depression and Anxiety after Acute Myocardial Infarction Treated by Primary PCI	2016	Prospective, observational study	Patients with ST-elevation MI (STEMI) treated with primary percutaneous coronary intervention (PCI) initially experience low levels of depression and anxiety, but these symptoms gradually increase over the course of a year after the procedure, highlighting the need for ongoing medical attention to address mental health.
Podolec et al. [88]	Depression in Cardiac Patients Is a Major Cardiovascular Event Risk Factor: A 12-Month Observational Study	2024	Prospective, observational study	Depression is prevalent among CVD patients and serves as a significant risk factor for one-year CVD mortality and adverse events, underlining the necessity for coordinated guidelines between psychiatry and cardiology societies to address this issue.
Seidl et al. [117]	The 3-Year Cost-Effectiveness of a Nurse-Based Case Management versus Usual Care for Elderly Patients with Myocardial Infarction: Results from the KORINNA Follow-Up Study	2017	Randomized, clinical trial	Nurse-based case management was cost-neutral and led to significant improvements in health status among survivors, with higher quality-adjusted life years (QALYs) and lower costs; however, the differences in both costs and QALYs were not statistically significant.

Prabhakaran D et al. [133]	Yoga-Based Cardiac Rehabilitation After Acute Myocardial Infarction: A Randomized Trial	2020	Randomized, clinical trial	Yoga-based cardiac rehabilitation (CR) improved self-rated health and the ability to return to pre-infarct activities after acute myocardial infarction, though it lacked statistical power to show a difference in MACE, suggesting it could be a viable alternative when conventional rehabilitation is unavailable or unsuitable.
Varnfield M et al. [141]	Smartphone-based home care model improved use of cardiac rehabilitation in postmyocardial infarction patients: results from a randomised controlled trial	2014	Randomized, clinical trial	The smartphone-based home care CR program improved post-MI CR uptake, adherence, and completion, and proved as effective as traditional CR in improving both physiological and psychological health outcomes, making it a viable option for optimizing CR service use.
Ferguson T et al. [144]	Effectiveness of wearable activity trackers to increase physical activity and improve health: a systematic review of systematic reviews and meta-analyses	2022	Systematic review	Wearable activity trackers effectively increase physical activity, improve body composition, and enhance fitness across various populations, with benefits sustained over time.
Castellao JM et al. [145]	Polypill Strategy in Secondary Cardiovascular Prevention	2022	Randomized, clinical trial	A polypill containing aspirin, ramipril, and atorvastatin taken within 6 months after MI significantly reduced the risk of MACE compared to usual care.

Abbreviations: CVD, Cardiovascular Disease; CR, Cardiac Rehabilitation; HRQoL, Health-Related Quality of Life; MACE, Major Adverse Cardiovascular Events; MI, Myocardial Infarction; PCI, Percutaneous Coronary Intervention; QALYs, Quality-Adjusted Life Years; QoL, Quality of Life.
